# The spider cuticle: a remarkable material toolbox for functional diversity

**DOI:** 10.1098/rsta.2020.0332

**Published:** 2021-09-20

**Authors:** Yael Politi, Luca Bertinetti, Peter Fratzl, Friedrich G. Barth

**Affiliations:** ^1^ B CUBE - Center for Molecular Bioengineering, Technische Universität Dresden, Dresden, Germany; ^2^ Department of Biomaterials, Max Planck Institute of Colloids and Interfaces, Potsdam, Germany; ^3^ Department of Neurosciences and Developmental Biology, University of Vienna, Vienna, Austria

**Keywords:** biological material, hierarchical structures, mechanical properties, optical properties, spider cuticle

## Abstract

Engineered systems are typically based on a large variety of materials differing in composition and processing to provide the desired functionality. Nature, however, has evolved materials that are used for a wide range of functional challenges with minimal compositional changes. The exoskeletal cuticle of spiders, as well as of other arthropods such as insects and crustaceans, is based on a combination of chitin, protein, water and small amounts of organic cross-linkers or minerals. Spiders use it to obtain mechanical support structures and lever systems for locomotion, protection from adverse environmental influences, tools for piercing, cutting and interlocking, auxiliary structures for the transmission and filtering of sensory information, structural colours, transparent lenses for light manipulation and more. This paper illustrates the ‘design space’ of a single type of composite with varying internal architecture and its remarkable capability to serve a diversity of functions.

This article is part of the theme issue ‘Bio-derived and bioinspired sustainable advanced materials for emerging technologies (part 1)’.

## 1. Introduction

Materials have always been a driver of technological progress in human history, from copper and iron to plastics, magnets and semiconductors. The downside of this development in the current climate crisis is that about 32% of all anthropogenic greenhouse gas emissions worldwide result from material production, more than half of which are due to steel and cement industries alone [1]. This calls for a wide-ranging effort to change production processes but also to rethink the way in which materials are conceived and used in the design of devices.

Traditionally, materials are characterized by their properties that are chosen for applications according to their property profile, as displayed for example in Ashby maps [2]. This led to the multiplication of materials with specific properties, each of them tuned to a given application. This diversity of material compositions is increasingly recognized as a sustainability problem, since among other difficulties, it impedes reuse in a different function and complicates separation into components and, therefore, recycling [3]. By contrast, natural materials are multifunctional and generally built based on a comparatively small selection of base materials: proteins, polysaccharides and a few minerals [4]. Organic building blocks are typically fibrous and based on cellulose and chitin, the two most abundant polymers on earth, and on proteins (for example silk, keratin or collagen). Inorganic components include silica, calcium carbonates, calcium phosphates and magnetite, for example [5]. A huge flexibility in structural organization allows organisms to achieve a wide range of materials properties and remarkably fine-tuned structural adaptations using these building blocks. Due to evolutionary constraints dictated by the phylogenetic history of the organism, multifunctionality emerged as a common solution. The exoskeletal cuticle of arthropods, based mostly on a composite of chitin filaments and protein, is a remarkable example of this versatility [6]. The cuticle is not just a skin-like protection, but represents the lever system needed for locomotion and acts as a support structure for numbers of tools for piercing, cutting and interlocking, for the reception, transmission and filtering of sensory information, for structural colours, transparent lenses, light manipulation and more. Unlike in human technology, this is all achieved by variations of the same theme based on identical building blocks, which enables the chitin–protein composite to locally acquire the properties needed for each of these tools [7]. In this way, the chitin protein material of the arthropod cuticle is able to fill large portions of an Ashby map without major variations in chemical composition. In this paper, we are reviewing the cuticle properties as found in spiders, a large order within the phylum of arthropods. The idea is not to suggest direct biomimetic translation of the systems described, but rather to show how by the variation of fibre architectures and a minimum of additives, a broad range of functionalities can be obtained with the same material basis. With the abstraction that fibres do not necessarily need to be made of chitin, the examples shown here could inspire new ways of generating multiple material properties and fine-tuned functional structures without increasing the diversity of material compositions.

## 2. Composition and structure of the spider cuticle

### (a) Composition

The cuticle is formed by a layer of epidermal cells that secrete building blocks into a small space called the assembly zone [8]. Once assembled the cuticle does not contain living cells. The building blocks used by spiders to build their cuticle are chitin, a linear polymer of *N*-acetyl-glucoseamine, proteins and glycoproteins, including the available 21 amino acids and multiple post-translational modifications of those, sclerotization agents (typically catecholamines) and water. Additionally, pigments, transition metal ions and halogens are sometimes deployed in a small amount.

**Figure 1.**
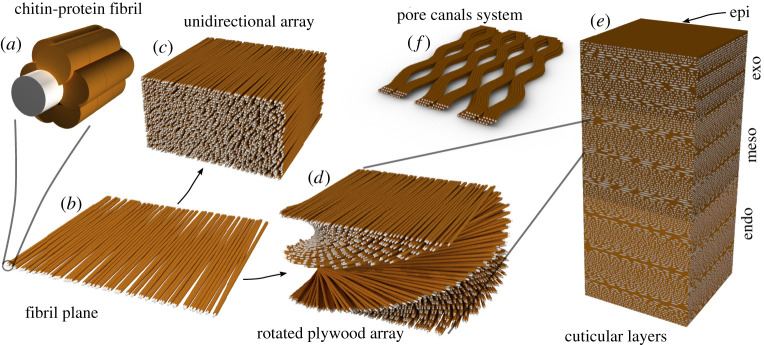
A schematic diagram of the hierarchical structure of spider cuticle. (*a*) The chitin–protein basic building unit. The grey core represents the chitin crystal, whereas the proteins are represented in brown. (*b*) The fibril sheets. Chitin–protein fibrils are arranged roughly in parallel. (*c*) Unidirectional arrangement of fibril sheets. (*d*) Helicoidal arrangement of fibril planes. (*e*) The cuticle layers made of layers of parallel and helicoidal arrays. (*f*) Pore-canals cross-sections, the channels transverse the entire thickness of the procuticle. (Online version in colour.)

In the procuticle (i.e. the part of the cuticle that contains chitin), chitin is arranged in the crystalline alpha form as 3 nm wide nanofibrils several hundreds of nanometres long [9,10] (figure 1). These are wrapped by an organized protein coat, forming a nanofibril that is the main structural component of the cuticle. In addition to chitin binding proteins, some structural cuticular proteins are not directly associated with chitin and may be considered as matrix proteins. The composition of the cuticular proteins may vary between different regions of the cuticle leading to variation in the matrix mechanical properties due to a direct effect of varying the protein–protein interactions, or secondarily by changing the propensity for cuticle hydration [11].

The arthropod exoskeleton is a hard shell that limits the growth of the animal. In order to increase in size, the spider goes through several moult cycles in which a new cuticle is built, folded under the old one that is then shed (ecdysis). The new exo-cuticle is then stretched and expanded by haemolymph pressure emanating from increased heart and muscle function [8]. The inner layers of the cuticle, endo and meso-cuticle continue to be synthesized for several days or several dozen days depending on the moult cycle. Several hours after ecdysis the new cuticle is sclerotized, a process in which the matrix proteins are covalently cross-linked by oxidation products of catecholamine [12]. The sclerotization process is also accompanied by drastic dehydration of the cuticle, which together lead to a stark increase in material stiffness. While other groups of arthropods, specifically crustaceans, which include for example, shrimps, crabs and lobsters, use calcium carbonate and calcium phosphate minerals to stiffen and harden their shells [13], spiders do not mineralize. They, however, use metal ions, such as Zn, Ca and Mn in order to cross-link specialized cuticular parts through metal ion coordination leading to increased hardness and stiffness of their fully organic cuticle [14,15]. Finally waxes, implemented in the epicuticle, make the cuticle water impermeable and allow the incorporation of pheromones.

### (b) Hierarchical structure

The chitin–protein fibres organization can be described as an arrangement of two-dimensional sheets in which the fibres are oriented roughly in parallel (figure 1). These sheets are then stacked on top of each other. The orientation of the fibres in each sheet is either unchanged or slightly rotated with respect to the sheet above or below [16]. The result is a parallel stack arrangement, akin to an aligned nematic organization of liquid crystals or a helicoidal structure akin to the cholesteric liquid crystal organization. In cuticular sample sections, the helicoidal arrangement gives rise to a lamellate appearance, in which every lamella represents half the pitch of the helicoid or the accumulated 180 degrees rotation of the fibres' long axes. The rotation angle in different parts of the cuticle is variable, leading to lamellae of varied thickness that can be used to form a cuticle with variable properties [16,17]. The cuticle is perforated by multiple pore-canals that span the entire thickness of the procuticle. These canals are used for example for the transport of sclerotization agent through the cuticle [8]. Water and air-filled porosity is also harnessed in order to support large mechanical strain or to produce structural colours as discussed below. At the highest level of hierarchy, the overall shape of an organ and its surface roughness are often modified in a way that supports a particular biologically relevant function.

**Figure 2.**
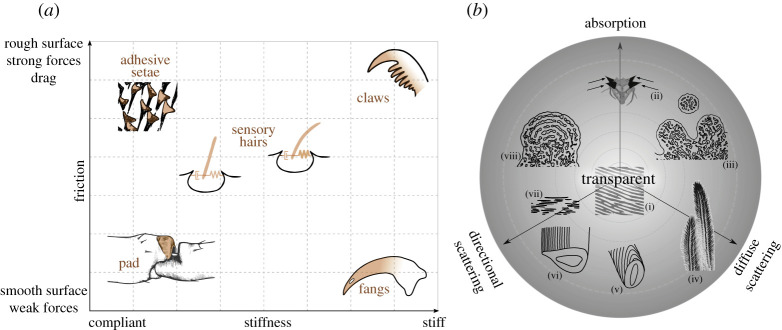
Design spaces. (*a*) Mechanical design space contrasting friction (surface property) vs. stiffness (bulk property). *Top left*: spatula ends of adhesive setae (*Cupiennius salei*). *Top right: C. salei* tarsal claw. *Bottom right: C. salei* fang. *Bottom left*: the cuticular pad in *C. salei* acts as a high pass filter for surface vibration in front of the metatarsal lyriform slit organ. *Center*: sensory hairs (setae); variations in spring constant stiffness and damping properties determine the hairs' sensitivity. (*b*) Optical design space. The design space is depicted as a projection of a cone along its central axis. The tip of the cone (centre of the circle (i) represents transparent properties. It is represented by anti-reflective epi-cuticlar nano-structures, as found on the cornea of spiders. Three axes emerging from the tip are: absorption, directional and diffuse scattering. The structures discussed occupy different regions within the design space. (ii) In the spider *P. rubroargentea*, the spiny cuticular extensions contain melanin within the cuticle as well as in melanophores. (iii) Schematic of cross-section of the yellow hairs of *P. metallica*, showing short-range order cuticular/air domains. (iv) Morphology of the yellow hairs of *P. metallica*, microtrichs contribute to diffuse scattering. (v) Schematic of the scale of *M. nigromaculatus*: diffraction grating on a curved surface. (vi) Schematic of the scale of *M. robinsoni*: diffraction grating on a flat surface. (vii) Guanine platelet crystals in the tapetum of spider eyes. (viii) Schematic cross-section of the blue hairs of *P. metallica*, showing order cuticular/air lamina. Note that the distribution of the structures along the depth of the cone is lost in this representation. (Online version in colour.)

In the following, we describe two multidimensional design spaces (figure 2) in which we contrast different material properties. First, we discuss the mechanical design space available to the spider to build its cuticle as shown by the remarkably fine structures, which serve a large spectrum of functions. The second design space covers the optical properties of the spider cuticle. The subsequent sections will exemplify various regions in these design spaces demonstrating the structural flexibility inherent to the material ‘design’ of the cuticle.

## 3. The mechanical design space

Figure 2*a* shows a two-dimensional diagram illustrating a mechanical design space relevant to the spider cuticle. We chose two axes that relate to the bulk and surface properties of the material. The abscissa represents the elastic properties of the material while friction or drag increases along the ordinate. While figure 2*a* is a schematic diagram with arbitrary units, Young's modulus in spider cuticle samples so far studied covers four orders of magnitude in the range around 0.1–20 GPa [18]. On either axis a modification of the material properties can be achieved by structural adjustments or by a change of the volume fraction and chemical properties of its components.

**Figure 3.**
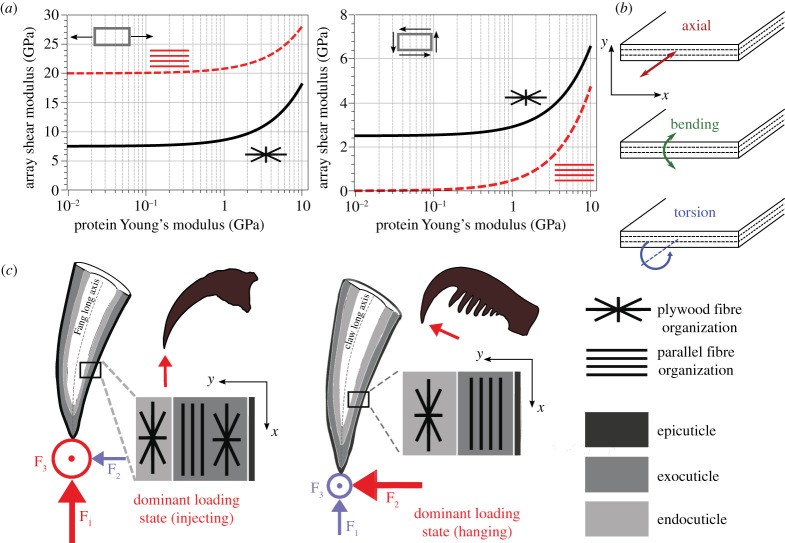
Mechanical properties of cuticle depend on the architecture of the fibre array. (*a*) Effect of the proteins’ Young's modulus on the array moduli. *Left*: Young's modulus. *Right*: shear modulus of parallel-fibred and rotated plywood fibril arrays. The estimated modulus of the chitin fibrils is 100 GPa; the modulus of the array was calculated considering a chitin volume fraction of 0.2. (Redrawn with permission from ref. [20]). (*b*) Possible mechanical loads: *Top*: axial tension. *Middle*: bending. *Bottom*: twisting, applied on a cuticle cross-section. In the schematic, the *x*-axis is parallel to the cuticle surface, the *y*-axis is oriented across the cuticle thickness. (*c*) Schematic illustration comparing the cuticle micro-architecture in the fang (left) and claw (right) of the spider *C. salei* in relation to their mechanical function. The dominant loads upon prey capture (fang) and attachment to rough surfaces (claw) are depicted in red, minor loads are depicted in blue. Reproduced with permission from ref. [15]. (Online version in colour.)

To a first approximation, the elastic properties of the cuticle material are dominated by the stiffness of the individual phases (chitin and proteins) and their relative volume fractions [16,18]. Chitin fibres are stiff along their long axis, with predicted Young's modulus around 100 GPa [19]. The modulus of the protein matrix, instead, is around 10–100 times lower, mainly depending on its degree of sclerotization. Using rules of mixture, it was shown that chitin dominates the axial stiffness of the chitin–protein fibril, while the protein dominates its perpendicular and shear stiffness [18]. Bar-On *et al*. [20] estimated Young's and shear modulus of fibre arrays with parallel or rotated plywood architecture as a function of the protein modulus (figure 3*a*). Evidently, the parallel arrangement provides higher axial stiffness to the array but compromises shear stiffness relative to the rotated plywood fibre array. Thus, a parallel array will resist axial load and provide bending stiffness but hardly resists twisting moments. The plywood array provides higher—between five times and two orders of magnitude—shear stiffness as compared to a parallel array [20]. It is, therefore, not surprising that the spider tarsal tendons are composed of parallel fibre bundles along a large part of their length [10]. The ventral tarsal tendon is pulled and relaxed by the attached muscles, which flex the tarsus and move the pretarsus with its claws, leading to predominantly axial forces [21].

The rotated plywood and the parallel fibre architectures are complementary from a mechanical point of view and combining them within the cuticle allows functional adaptations addressing different mechanical demands [18]. The axial and bending stiffness of the layered structure are defined as the sum of the products of the axial Young's modulus and the array's cross-sectional area or the moments of inertia of the layers, respectively. The twisting stiffness is related to the array's shear modulus and the polar moment of each layer (figure 3*b*) [22]. Importantly, the moments of inertia and the polar moments depend on the location of the array within the material cross-section. This means that while the axial stiffness only depends on the cross-sectional area of the structured layer, both the bending and twisting stiffness will be influenced by the specific localization of a particular structured layer within the cuticle section [18]. Specifically, the outer layers will dominate the bending and twisting stiffness of the cuticle as compared to inner layers [22]. The inner layers are assumed to protect against buckling. In the spider's claws and fangs described below, the corresponding organization of layers seems to reflect this constraint.

The fracture mechanics of the cuticle is governed by various toughening mechanisms across the structural hierarchy [23] that include multiple interfaces such as chitin–protein and protein–protein interactions, interactions between fibrils, between layers with different architecture (e.g. parallel and rotated plywood), or different composition (e.g. endo-, exo-, epi-cuticle), which will not be discussed here. At the microstructural level, however, the rotated plywood structure has huge implications for the cuticle fracture mechanics. The rotated plywood structure is widespread in biology. Its mechanical behaviour has been extensively studied in bone [24,25] and arthropod cuticle [16,26]. A crack propagating through a stack of lamellae with fibres arranged as rotated plywood proceeds in either a direct or spiral path. In the first case, crack orientation is preserved through successive layers with fluctuating changes in the effective modulus leading to oscillation in the fracture driving force [18,27]. In the spiral-path mode, on the contrary, crack propagation occurs by separating the interfaces between adjacent fibres without breaking them. This leads to periodic alteration of the crack tip propagation direction, significantly increasing the length of the crack path and its surface area per volume. The energy required to fracture the material is increased [28]. Furthermore, the development of microcracks ahead of a crack tip was recently shown by finite-element simulations using a two-dimensional model for a material with periodically varying mechanical properties. The formation of these microcracks in turn enhances energy dissipation and therefore the toughness of the array [25]. Multiple pore-canals in the cuticle further lead to stopping or deviating cracks [18].

Finally, the external most layer of the cuticle, the epicuticle, contains no chitin fibres and is typically hydrophobic. The intrinsic properties of the epicuticle are tuned by the degree and type of matrix cross-linking. Despite its small dimension, the epicuticle serves multiple mechanical functions. It may be smooth or show surface roughness at multiple length scales. This roughness typically is due to miniature ‘hair-like’ extensions or elongated ridges. Such surface structures, which are common throughout the arthropods, lead to various effects including anti-reflection, as discussed below, the modulation of wettability and hydrophobicity, self-cleaning, antibacterial and antifungal protection and defence (reviewed in [29]).

Below, we describe several examples found in spider cuticle that illustrate the material properties in different regions of the mechanical design space (figure 2*a*). We start by structures at the upper left corner—the *adhesive setae*, which are sticky and compliant. We then describe the spider *claw*, which is rather stiff and used for the attachment to the substrate by interlocking. The spider *fang* is an example of a stiff appendage that employs a smooth surface reducing friction. Accordingly, it occupies the bottom right corner of the design space. The *hair-like structures* that are used by some spiders to trap air bubbles are superhydrophobic and compliant. Finally, we focus on the auxiliary structures of the spider's highly sensitive *mechano-sensors*. These are rather intricate and include multiple sub-structures with varying mechanical properties and are all made of the same basic cuticular material. The specific organization of these sensory structures promotes the biomechanical filtering of the sensory stimuli before they are transmitted to the nervous system. Thereby information is pre-processed mechanically, the central nervous processing simplified and its energetic cost reduced, and the sensory focus shifted to the biologically relevant stimulus patterns [30].

### (a) Soft and sticky: spider attachment setae

**Figure 4.**
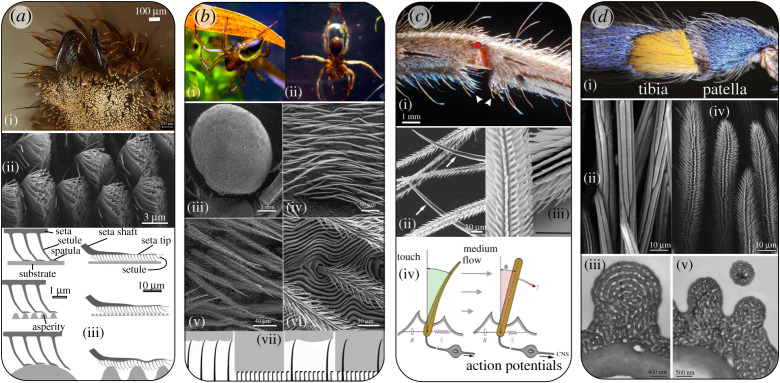
Spider setae for multiple functions. **(*a*) Attachment**: Adhesive setae. (i) Spider attachment claw tuft in *Cupiennius salei*. Light micrograph courtesy of Dr. Maryam Tadayon. (ii) Adhesive setae of the spider *C. salei*. The setae extend microtrichs ending with a flat spatula. Photo: Jonas O. Wolff and Benjamin Eggs, with permission of the authors Ref. [107]. (iii) Schematic illustration of the hierarchal arrangement of adhesive setae during their attachment to surfaces with different roughness. Image adapted with permission from ref. [34]. **(*b*) Respiration**: Plastron in the spider *Argyroneta aquatica*. (i) Air clinging to the hydrophobic hairs of the spider opisthosoma. (ii) A large bubble, captured at the water surface and held by the hairs on the opisthosoma and rear legs. Image adapted with permission from ref. [44] (iii) SEM micrograph of opisthosoma of the spider *A. aquatica*. (iv–vi) increasing magnification of the setae of the opisthosoma. Images used with permission from ref. [108]. (vii) Schematic illustration of the air captured by long and short microtrichs on physical gill setae. *Left*: a large air volume (white) is trapped between the water (grey) and the surface. *Left,middle*: a surface with small and dense microtrichia capturing a small volume of air that is retained for long time. *Right, middle*: a surface with both long and short setae/microtrichia. A large volume of air is trapped. *Right*: when water pressure is high, the air bubble reduces its volume. The microtrichia are in contact with the water, the small air volume is retained for a longer time (reproduced with permission from ref. [43]). **(*c*) Mechanoreception**: Hair-like mechanosensors in the spider *C. salei*. (i) The tibia-metatarsus joint of a second walking leg. The red dot marks the centre of rotation of the joint. Arrowheads point to joint proprioreceptive hairs. (ii) Surface morphology of sensory hairs in *C. salei*. The arrows point to two smooth setae. Reproduced with permission from ref. [109]. (iii) interlocking microtrichs on the hair shaft of proprioreceptive hairs sown in (i). Images in (*c* i,iii) are reproduced with permission from ref. [56]. (iv) A schematic of arthropod touch (left) and medium-flow sensitive hair sensilla (right); *R* viscosity, *S* elasticity, CNS: central nervous system, θ deflection angle. Arrows with different lengths indicate difference in medium-flow velocity due to boundary layer. [47, modified] **(*d*) Coloration**: (i) Blue and yellow setae of the spider *Poecilotheria metallica*. (ii) SEM micrographs of the surface of the blue (ii) and yellow (iv) setae seen in (i). TEM micrograph of a cross sections of the blue hair (iii) and the yellow hair (v). The innermost layer of the seta shaft is solid; the outer layer consists of six regular lamellae (1–6), alternating with air spaces in the blue hair (iv). In yellow hairs an irregular cuticular meshwork occupies the peripheral part of the hair shafts (v). Reproduced with permission from ref. [81].

Adhesive setae are common in wandering spiders, but are absent in species that build silken webs [31] (figure 4*a*). These setae are used to secure the spider onto smooth surfaces during rest, locomotion and prey capture [32,33]. They can be found in high density (e.g. over 2000/mm^2^ in *Cupiennius salei*) at the claw tufts (so-called tarsal attachment scopulae) or at other parts of the spider legs [34]. The setae are built hierarchically. They are asymmetric; the surface of the seta facing the substrate contains multiple setules terminating as a flat spatula, giving rise to a two-dimensional ‘hairy’ system (figure 4*a*(ii)) [34]. The multiple contact sites, the compliance of the setae and their spatial arrangement allow the spatial adjustment of the attachment tufts to the roughness of the surface and provide damage resilience upon retraction. Spiders rely on dry attachment mechanism, more like geckos than insects. Attractive forces are gained by the short-range intermolecular van der Waals interactions [35]. Therefore, high compatibility with the surface structure is crucial for achieving large contact area leading to strong adhesion.

The setae and their spatulae are made of cuticle. Here, the stiffness of the chitin–protein fibres provides tensile strength upon detachment [36,37]. The thickness of the spatulae (around 30 nm) renders it flexible enough to achieve a large contact area with the nanometre scale roughness of the surface. At higher surface roughness, in the order of several micrometres, the attachment forces are greatly reduced due to the reduction of contact area. It is suggested that the reduction in attachment force of single setae at high roughness levels is advantageous in preventing self-matting; as the surface roughness of adhesive setae at the opposite side from the setule is in the length scale of the critical roughness size, sticking of neighbouring setae is prevented [34].

### (b) Stiff structures with high and low friction: claws and fangs

As surface roughness increases, spiders use their claws in addition to the adhesive setae to keep themselves attached to a surface. Due to their curved grapnel-like shape and high stiffness, the claws promote attachment by interlocking to surface roughness. In the spider *C. salei*, the epicuticle on the active, concave (ventral) side of the claw is thickened and shows surface irregularities at various length scales [15]. These increase the friction with the surface and secure the grip [38]. However, the rough epicuticular surface is also more prone to wear than a smooth surface would be. This problem is mitigated by an increased wear-resistance of the cuticle at the ventral claw surface as compared to the base of the claw. This is achieved by the incorporation of a small amount (approx. 1.5 at. %) of Mn and Ca ions that act as cross-linkers in the proteinaceous matrix, leading to abrasion tolerance that is similar even to some highly mineralized matrices such as enamel and nacre [15].

The overall stiffness of the claw is achieved by its fibre architecture. Remarkably, the exo-cuticle in the large part of the claw toward its tip shows parallel fibre layers oriented along the grapnel long axis, whereas the endocuticle is composed of fibres arranged in the rotating plywood manner. It is instructive to compare the fibre architecture in the claws of the spider *C. salei* with that in its fangs. The fangs are used to inject venom into the spider's prey. They need to puncture through the prey's cuticle, typically made of similar chitin–protein based material. In the fang the parallel fibre organization is sandwiched between two rotated plywood layers (figure 3*c*) [14]. Both the claws and the fangs accommodate significant bending loads during their natural function, but the fangs also have to accommodate twisting loads not expected in the claws. The forces generated in the claw are mainly within the plane of the claw, while biting-induced load of the fangs can in addition generate significant non-planar forces [39]. As mentioned above, the localization of layers with specific architecture in the layered composite has a strong effect on the materials bending and twisting stiffness (figure 3). Thus, the overall microstructure of the spider claw seems to be adapted to resist bending-dominant loads, whereas the alternating plywood/parallel fibre arrangement in the cuticle of the fang may be viewed as an adaptation to resist combined bending–torsion loads [15,18].

In addition to the refined fibre arrangement, the protein matrix in the fangs of *C. salei* is also enriched with metal ions. Zn ions in a concentration of up to approximately 6 at. % in the fang's tip promote local hardening and stiffening of the cuticle by cross-linking with histidine residues [14,40]. Interestingly however, abrasion resistance in the fang is comparable with that in the Mn and Ca enriched claw, despite the fact that the former contains more than three times the amount of metal ions [15]. The smoothness of the surface of the fang tip suggests that friction is reduced as compared to the ventral surface of the claw [41]. The smooth surface will allow the spiders to use their fangs to repeatedly puncture prey cuticle and to retract the fangs, as observed during prey capture and feeding. The fang and claw examples demonstrate that small architectural modifications in the layered structure as well as the surface structure of the cuticle support two different mechanical requirements.

### (c) Soft and hydrophobic: plastrons and physical gills

Some spiders (e.g. *Dolomedes fimbriatus* and *Argyroneta aquatica*) take air bubbles under water that serve as primary O_2_ source and as physical gills obtaining O_2_ from the water [42]. The air layer is maintained on superhydrophobic air-retaining surfaces composed of multiple setae, often with multiple microtrichs on their surfaces (figure 4*b*). The length and density of the setae vary largely between species. (i) Large and sparse arrays of setae can retain a large air bubble, but the bubble is only loosely supported and will quickly reduce its volume. (ii) Small and dense arrays of hairs retain the air for a longer time, even days and weeks. They form a plastron, that is a stable, incompressible thin air layer [43]. The diving bell spider, *A. aquatica*, weaves a silk web dome underwater, attached to submerged vegetation. Performing repeated trips to the surface, it then fills the dome with air retained in a large bubble held by multiple cuticular setae on the ventral side of its opisthosoma and its hind legs (figure 4*b*(ii)) [44]. The setae are long and contain multiple microtrichs along their entire length rendering them superhydrophobic. Due to the large size of the silk supported bubble the surface area sufficiently supports the oxygen influx needed for the spider's metabolism at rest. The setae on the opisthosoma (figure 4*b*) hold a stable plastron that supports short prey capture excursions or trips to the water surface [44].

### (d) Mechanical gradients and interfaces: mechanoreceptors

In the context of mechano-sensing, mechanical gradients in the cuticle material are central to the biomechanical filtering and transmission of stimuli. Specifically, the cuticular non-nervous auxiliary structures of mechano-sensors closely associated with or even fully embedded in the exoskeleton play a significant role in the animal's biology. Biological sensors and nervous systems are energetically costly [45]. Passive biomechanical pre-processing of stimuli may have offered a large advantage when sensors evolved, if indeed saving energy had been a dominant evolutionary selection pressure in the evolution of sensors [46]. The optimization of cuticular structures such as setae or sensory slits for the selective uptake of the relevant stimuli by adjusting sensitivities and working ranges will entail reduced energy consumption as compared to a more exclusive stimulus processing by the nervous system. Spiders demonstrate impressively how small morphological and micro-architectural modifications of the cuticular auxiliary sensory structures enable the transmission and transformation of the biologically relevant stimuli and the filtering out of other stimuli and noise. Learning how to achieve this in engineering may lead to a substantial decrease of the computational power needed for data processing and thus reduced energy consumption.

#### (i) Hair-like sensors for touch and medium flow

The exoskeleton of many spiders is covered with hair-like structures. Most of those setae are innervated and have a sensory function. Deflection of the hair shaft elicits a neuronal response. The hair is suspended by a membrane within a socket. One or several bipolar sensory cells are coupled to the base of the hair by their dendrites. In spiders, hair-like mechano-sensors are either air flow sensors (trichobothria) or tactile hairs (setae) [47]. The length and surface structure of the hair shaft, its material properties and the properties of the supporting membrane largely determine the mechanical sensitivity and stimulus specificity of the sensory setae.

***High sensitivity and large dynamic range.*** The spiders' air flow sensitive trichobothria are among the most sensitive sensors in the animal kingdom. The hair is deflected by the tiny frictional forces (as low as 0.4–4 × 10^−6 ^N [30]) exerted by particles in the surrounding medium (air), and work close to the limit of thermal noise [47]. By contrast, the minimum force needed to elicit a response in tactile hairs is considerably larger, around 5 × 10^−5 ^N/° [30].

Primarily, the outstanding mechanical (and consequent physiological) *sensitivity* of the trichobothria is determined by the properties of their suspension, specifically the torsional restoring constant (the spring stiffness, *S*) and its viscous damping (*R*) (figure 4*c*). These are extremely low in the air flow sensors as compared with tactile hairs. For *C. salei*, the spring constant is four orders of magnitude lower than that of its tactile hairs (setae). The values for the damping constant, *R*, are extremely low as well (in the order of 10^−15 ^Nm s/rad) [48,49]. The physical structure acting as a spring most likely is the suspending membrane in the socket, whereas the damping properties are also attributed to the fluid flow of the receptor lymph surrounding the base of the hair [50]. The suspension membrane is an extension of the cuticle and made from material similar to that of the soft joint membranes, which typically have a high chitin volume fraction (about 50%), are highly hydrated and not or hardly sclerotized. Unfortunately, however, due to their small dimensions, it is unknown to date how the particular properties are achieved. It is postulated that elastomeric proteins such as resilin, found in insect cuticles, or elastin, found in vertebrate connective tissues, contribute to the membrane viscoelastic properties [50].

Because of the much higher resistance of the tactile hair (setae) suspension to hair deflection (as compared to the trichobothria), the hair shaft bends when deflected. The bending point, however, is shifted down towards its base with increasing loads from above, thereby shortening the effective lever arm. As a result, the hair is less susceptible to breaking and its working range is extended. Most importantly, this leads to higher mechanical sensitivity for smaller loads (as at stimulus onset) rather than for larger ones. The shift of the bending point with increasing load results from the increase in cross-sectional area along the hair length (from tip to base) and with it the second moment of area. The hair (setae) shaft is postulated to be a structure of uniform maximum strength in regard to axial stresses that are the major ones during bending [51].

***Stimulus specificity*.** Despite the high sensitivity of their sensory hairs (setae), spiders can distinguish biologically relevant stimuli from background noise [52]. Background air movement during the spider's nocturnal activity is dominated by low frequencies (less than 10 Hz) and low flow velocities (less than 0.1 m s^−1^), whereas an effective prey stimulus contains a broad frequency range that extends above 100 Hz and highly fluctuating flow velocities of up to 1 m s^−1^ (reviewed in [53]). The hair shaft suspension shows viscoelastic behaviour. The torque required to deflect the hair at low angular velocities (of the order of 10^−4^ rad s^−1^), is around 3 × 10^−14 ^Nm, about half of that needed at higher angular velocities (of the order of 10^−1 ^rad s^−1^) supporting the phasic nervous response of the trichobothria to low angular velocities, occurring at the onset of hair motion from rest [47,54].

The length of the hair shaft of the trichobothria in *C. salei* varies between *ca* 100 and 1500 µm, which correlates with their frequency tuning in the range of around 40 Hz to more than 600 Hz. Whereas the lower frequency sensitivity limit is set by the width of the boundary layer, which increases with decreasing medium-flow frequency, the upper limit is set by the inertia of the hair shaft, which increases with hair length and air flow frequency [55]. Spider trichobothria typically occur in clusters of 2–30 hairs with varying length, which then form sets of bandpass filters enlarging the group's overall range of high sensitivity at a particular location of the exoskeleton [53].

***Directional sensitivity.*** Notwithstanding the morphological details of the dendrite attachment to the hair (setae) base, the shape of the cuticular socket in which the hair is suspended determines the directions in which the hair can be deflected, leading to directional sensitivity. In addition, heterogeneity of the hair's membranous suspension leads to a directional dependency of the torque resisting deflection that can even be smaller by about two orders of magnitude in the direction of natural stimulation (*ca* 5 × 10^−12 ^Nm/°) compared to other directions [56].

***Hair hierarchical structure: microtrichs for friction and drag increase.*** Many tactile setae and trichobothria show a feathery structure with multiple cuticular projections (microtrichs). The tips of microtrichs on the shaft of proprioreceptive hairs at opposite (facing) sides of the tibia–metatarsus joint form miniature hooks (figure 4*c*) leading to reversible interlocking when the joint flexes during locomotion. The enhanced contact between two interacting hairs, therefore, ensures reciprocal hair deflection eliciting a nervous response [56]. In the trichobothria of *C. salei*, microtrichs increase the drag exerted by the air flow. The spaces between the microtrichs are in the order of the boundary layer thickness when exposed to viscous (low Reynolds number) medium flow. The air flow in between the microtrichs is, therefore, arrested, thereby turning the feathery hair shaft into a paddle, effectively increasing hair diameter without a corresponding increase in hair mass [49].

#### (ii) Spider strain detectors—cracks, pores and channels

Spiders sense cuticular strain by their slit sensilla [46]. These are elongated (length *ca* 8–200 µm) narrow openings (width 1–2 µm) embedded within the exoskeletal cuticle. A thin cuticular membrane, to which the dendrite of a sensory cell attaches, covers them at the outside. At the inner side the slit is closed by a thin membrane as well, close to which the dendritic end of a second sensory cell is found [46]. The slits are most easily compressed by loads perpendicular to their long axis leading to highly directional mechanical sensitivity [46]. The sensitivity of an individual slit increases with its length. Many slits are arranged in parallel arrays of up to 30 slits, called lyriform organs. The location of a slit within the array greatly affects its compression response to cuticular strain, as well as the overall mechanical sensitivity of the array [57–60]. Depending on the type of array and slit distribution (lateral shift of slits, length gradation, orientation, etc.) the compression of an individual slit in the array may be reduced or amplified relative to that of an isolated slit of the same shape [59] (reviewed in [46]).

**Figure 5.**
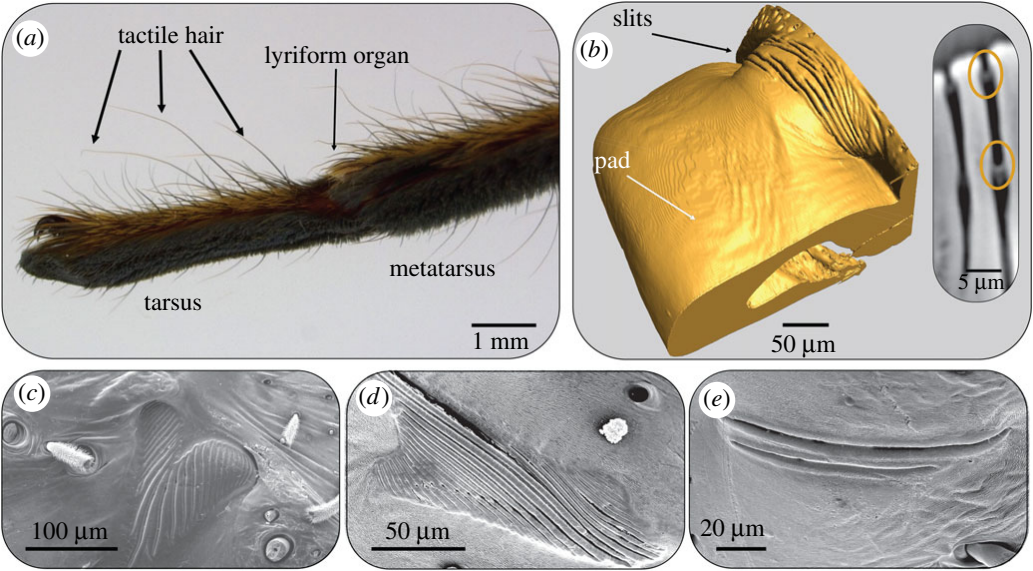
Spider strain sensors—cuticular slits in the spider *C. salei*. (*a*) The vibration sensitive lyriform organ HS10 is placed at the joint between the tarsus and metatarsus. Light micrograph courtesy of Dr Maryam Tadayon. (*b*) Micro-computer tomography (microCT) reconstruction of the lyriform organ HS10 and the adjacent cuticular pad. Inset: a virtual section through the reconstructed microCT data showing the outer and inner membranes. (*c*–*e*) Slit arrangements in different lyriform organs on the walking legs of *C. salei*. Reproduced with permission from ref. [46]. (Online version in colour.)

In the case of the metatarsal lyriform slit sense organ on the most distal leg joint (figure 5*a*), a cuticular pad distal to the slits (figure 5*b*) serves as a high-pass filter and a bumper protecting the slits against damage from over compression. In addition to sensing substrate vibrations the metatarsal organ also serves as a proprioreceptive sensory organ during spider locomotion with intriguing consequences. Substrate vibrations originating from predators, prey or courting partners lead to minute up and down movements of the tarsus that in turn presses against the pad. These vibration stimuli typically contain relatively high frequencies (*ca* 40 to several hundred Hz) and are sensed by the spider with extraordinary sensitivity [61,62]. Stimuli originating from the spider's locomotion are of low frequency (0.1–40 Hz) and sensed with much lower sensitivity [61]. Taken together, the range of vibration frequencies detected by the slits (though with strongly differing sensitivity) spans four orders of magnitude (figure 5).

The viscoelastic properties of the pad's unusually thick epicuticle alone explain the observed mechanical high-pass filter properties of the pad, which are reflected by the organ's physiological responses (tuning curve) as well [63,64]. But it is the structural arrangement of the pad's bulk that determines the transmittance of high-amplitude low-frequency proprioreceptive stimuli and protects the sensory organ against over compression and damage. The distal end of the pad is highly hydrated and shows a low elastic modulus (100 MPa) as compared to the proximal pad region (*ca* 10 GPa). Multiple fluid filled channels within the distal end of the pad were suggested to contribute to the pad's swelling and damping properties [65]. The gradient mechanical properties of the pad (soft distally, stiff proximally) are governed by cuticle hydration gradients that are in turn determined by a variation in lamella thickness and increased porosity. The structural organization of the cuticular pad and the properties of its epicuticle thus enable the multifunctionality of the metatarsal lyriform organ acting as the main vibration sensor of the spider as well as a proprioreceptor with ‘optimized’ sensitivity.

## 4. The optical design space

Figure 2*b* depicts an optical design space as a projection of a three-dimensional cone along its axes; transparency is situated at its tip (i.e. in the centre of the circle) from which three axes are extended to represent absorption, diffuse- and directional-scattering. The examples below demonstrate cases in which spiders use their cuticle to achieve various optical properties within this design space. From a functional point of view, spiders use colours for intraspecies communication, crypsis, thermoregulation, mimicry and aposematism [66].

### (a) Transparency

Typically, spiders have eight camera-type eyes. The number of eyes and their visual capabilities vary enormously with the spider's lifestyle, habitat and ecology. Vision is most often studied in jumping spiders (salticids) whose behaviour is largely guided visually. Their photoreceptor cells are at the focus of the lens and at least in some species spatial acuity, which is crucial for shape and form perception, has no rival among the insects and comes indeed close to that of primates and our own human eyes [67,68]. This differs from the eyes of web spiders, whose behaviour is dominated by mechanoreception. In jumping spiders, the two so-called primary eyes are dedicated to high resolution imaging, whereas the so-called secondary eyes are mainly motion detectors together providing large horizontal fields of view close to 360° [67–69].

**Figure 6.**
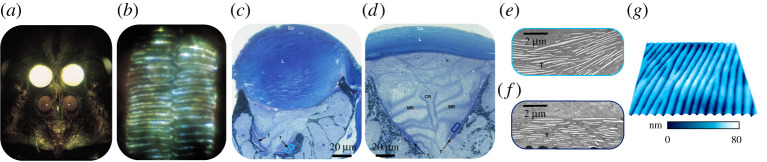
Spider eyes. (*a–b*) Eyes of *Cupiennius salei*. (*a*) Photograph of the primary and secondary eyes, showing the strong light reflection by the tapetum of the (upper) secondary eyes. (*b*) Seen through an ophthalmoscope through the transparent cuticular lens. the horizontal rows of photoreceptor cells are illuminated by light reflected by the tapetum. *a* and *b* reproduced with permission from [68]. (*c*) Methylene blue-stained cross sections through the postero-median eyes of the spiders (*c*) *Agelena labyrinthica* and (*d*) *Drassodes cupreus*. *c* and *d* reproduced with permission from [72]. (*e–f*) Electron micrographs of guanine crystals in the tapetum. (*e*) *Agelena labyrinthica* (*f*) *Drassodes cupreus*. Reproduced with permission from [72]. (*g*) 3D representation of corneal nanostructures in the eyes of a spider. Reproduced with permission from [73]. (Online version in colour.)

The highly transparent cornea and lenses of the primary eyes of jumping spiders allow almost 100% light transmission in the spiders' visual range, which extends from UV (around 300 nm) in some species, up to around 700 nm [70,71]. The cornea and the lens of spider eyes are made of lamellated cuticle (figure 6). In the few cases studied, the lamella thickness in the cornea is smaller than in the lens, in the order of 100 nm [72]. The typical pore-canals of the exoskeleton are absent in both cornea and the lens. Nano-scale roughness at the surface (cornea epicuticle) contributes to refractive index matching leading to an anti-reflectance effect similar to that of moth and butterfly corneas [73] (figure 6*g*).

### (b) Constructive and diffuse scattering and reflection

Constructive and diffusive scattering structures in spiders serve vision as well as the production of colour. In many spiders, a tapetum made of guanine crystals multilayers is located behind the photoreceptor cells of the spider secondary eyes. The tapetum reflects transmitted light back to the retina, increasing the amount of light harvested (figure 6). In the spider *Drassodes cupreus* that uses skylight polarization for navigation, the tapetum is V-shaped with the reflecting guanine multilayers oriented roughly orthogonally to each other (figure 6*d*), thereby increasing the polarization of the reflected light [72]. These eyes lack the typical corneal lens, and their cornea acts as a mere transparent window. Indeed, this spider's activity peaks shortly after sunset, when the skylight is particularly strongly polarized [72].

Guanine reflectors are also found in some spiders (e.g. *Tetragnatha montana*, *Thwaitesia argentiopunctata*) that show highly reflective silver shine on their opisthosoma. This broadband reflection is achieved by the organization of crystalline guanine platelets located intracellularly in specialized cells, guanophores, just beneath the hypodermis [66,74,75]. Similar to the tapetum, these guanin crystals are thus not an integral part of the cuticle and the reflection is only seen in areas where the cuticle is transparent. Interestingly, the guanine crystals in the guanophores of the spider *Latrodectus pallidus* are randomly packed and they display a prismatic habit leading to diffuse scattering and matte-white appearance [74]. Although not known in spiders, whiteness can also be achieved by cuticular structures. In the beetles *Cyphochilus* and *Lepidiota stigma* whiteness is achieved by a thin disordered anisotropic meshwork made of cuticular material that acts as a dense scattering medium [76–78].

The most striking structural colours are found in theraphosids (tarantulas) and salticids (jumping spiders). They produce structural colours by a variety of optical mechanisms. These include multi-layered cuticular structures [79,80], diffraction gratings [81–83], cylindrical Bragg mirrors [84], and combinations thereof. The result is iridescent and reflective structures with a wide spectral range including ultraviolet, blues and greens and even yellow, which is otherwise typically achieved by pigmentation [81]. The organization of cuticular material around voids that contain air, at the length scale of visible light wavelengths, gives rise to coherent or incoherent light interference resulting in perceived colour. Whereas whiteness can be achieved by diffuse (incoherent) broadband light scattering by uncorrelated disordered nanostructures, two- or three-dimensional periodic structures can select for a narrow bandwidth reflectance with strong iridescence [85]. Between these extreme cases a varying degree of disorder at various length scales broadens the reflectance bandwidth or reduces iridescence.

The different optical properties are obtained by producing three-dimensional structures of materials with different refractive indices (RIs). In fact, it is the RI contrast between the components forming the functional structure that determines the scattering efficiency. The RI values of the cuticular scales of the butterfly *Graphium sarpedon* are 1.57 and 1.54, at wavelengths 400 and 600 nm, respectively [85,86], considerably higher than the RI of air (approx. 1). Similarly, the RI of guanine is in the order of 1.83 in the direction perpendicular to the plane of the crystal platelets contrasting to the cytoplasmic spaces in gonophores for which the RI is estimated to be around 1.34 [82].

#### (i) Exploiting the helicoidal arrangement of the cuticle

Many insects, especially beetles, are known for using the helicoidal structure of the integument to produce vivid iridescence [87,88]. The iridescent colourful chelicerae of some salticid spiders are obtained in a similar manner where the multi-layered body cuticle leads to light interference [79,81]. An interesting case is found in the cuticle of the jumping spider *Cosmophasis thelassina*. Here, the multi-layered exo-cuticle is arranged with increasing pitch of the fibre lamellae from outside-in leading to a broadband light reflectance. However, epicuticular nanostructures on top of the cuticle form a diffraction grating with a period in the order of 460 nm that reflects blue light, leading to an overall yellow appearance for human vision [80].

#### (ii) Hairy colours: hierarchical cuticular photonic structures

Reflective and diffusive structures in spiders are often formed by cuticular material in hair-like structures (setae) and scales as discussed below. The bright blue colours of some theraphosid spiders (tarantulas) are due to specialized setae [81]. Here, a layered cuticular wall alternates with air layers causing light interference. In the spider *Poecilotheria metallica*, the surface of the blue hair forms elongated smooth ridges. The same spider shows bright yellow setae adjacent to regions of blue hairs (figure 4*d*). Morphologically these are similar to the blue hairs, but instead of having a regularly layered structure, their wall forms a short-range ordered cuticular meshwork of pores/struts with dimensions varying around 140 nm. Additionally, instead of being smooth the surface of the hairs has spiny extensions. The presence of these surface extensions and the long-range disorder of the meshwork lead to an angle-independent yellow appearance of these setae in contrast to the iridescent blue ones. Both hair (seta) types are transparent in transmission light attesting to the structural origin of the colour [81]. In some spider species, e.g. the blue fang tarantula, *Ephebopus cyanognathus*, the setae reflecting intense blue show a solidly pigment-filled core, thereby fully absorbing the non-reflected light. A similar effect is observed in wings of many butterfly species, where black scales underneath the coloured ones lead to an intensified colour appearance [89].

Blue and green reflectance originating from seta nanostructure have evolved and been lost multiple times in the evolution of theraphosids [90,91], leading to a diversity of nanostructures reflecting similar wavelengths. This again neatly demonstrates the inventiveness of nature and the remarkable flexibility of the cuticular material.

#### (iii) Cuticular scales: multilayers and diffraction gratings

Colours in jumping spiders are produced by scales. Scales are flattened setae lying parallel to the surface of the cuticle. The cuticle of some of these scales is enriched with pigments, as discussed below, but the colours of others are due to the structural organization of the scales at the nano- and micro length scales [81,92]. Some scales exhibit multilayer lamellae while others are simpler with two thin cuticular layers only surrounding a narrow air space [92].

The male of the jumping spider *Cosmophasis umbratica* reflects ultraviolet in addition to green-orange colours (wavelength around 600 nm) from scales showing cuticle-air-cuticle sandwich structure. Here, the cuticle layers are three-quarters of a wavelength thick and the air gap one quarter wavelength. This organization results in two reflectance peaks: one at around 600 nm and another at approximately 385 nm. In other scales of the same spider species, a similar cuticular thick layer lacking the air gap produces a purple reflection [93]. The blue scales of the peacock spider *Maratus splendens* show a sandwich structure similar to that of the green-orange and UV reflecting scales of *M. umbratica.* Differently, the inner side of the scales is covered by cuticular filaments that have been shown by modelling to significantly affect the reflectance spectra [92]. Obviously, spider scales show fine tuning of spectral reflectance that relay on only minor architectural changes.

**Figure 7.**
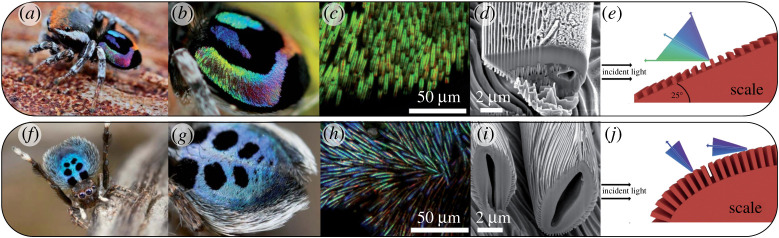
Peacock spiders with coloured opisthosoma. (*a,f*) Adult male (*a*) *Maratus robinsoni*, (*f*) *Maratus nigromaculatus*. (*b,g*) Close-up of the opisthosoma of (*b*) *M. robinsoni*, (*g*) *M. nigromaculatus*. (*c,h*) Optical micrographs of the opisthosomal scales showing the scale alignment in (*c*) *M. robinsoni*, (*h*) *M. nigromaculatus*. (*d,i*) SEM micrographs showing the scales' surface grating structure and the cross-sectional shape of the scale. (*d*) In *M. robinsoni* the grating is arranged parallel to the long axis of the scale with a mean period of ∼330 nm (*i*). The grating of *M. nigromaculatus* scales curves along the cylindrical scale. (*e,j*) Schematic depicting the reflection mechanism for both scales. Images reproduced with permission from ref. [82]. (Online version in colour.)

The scales of many jumping spiders show fine ridges on the surface acting as a diffraction grating. Some of the most striking examples are found in the Australian peacock spider *Maratus robinsoni* (figure 7). It shows a wide range spectral iridescence (red-to-violet to human vision) where each colour is displayed with high purity, whereas typically natural iridescent structures show a narrow hue reflectance range [83]. The teardrop shaped cross-section of the scales is covered on its prismoidal slope by a dense parallel binary-phase grating structure with 330 nm periodicity (figure 7) [82]. The combination of the parallel grating on top of the prismoidal slope gives rise to a static microscopic—reverse order—rainbow pattern at each individual scale. A similar albeit denser (period about 220 nm) grating is found in the scales of *M. nigromaculatus*, which are, however, cylindrical and predominantly reflect blue light with a robust angle-independent hue [82]. Furthermore, the scales are arranged in well-ordered parallel arrays on top of the opisthosoma in *M. robinsoni*, whereas in *M. nigromaculatus* the scales arrangement only shows a short-range order. The organization of scales at the macroscopic level further affects the degree of angular dependence of the visual appearance of these spiders (figure 7).

### (c) Colour by light absorption

Light absorption by the cuticle is often related to the presence of pigments and to cuticular tanning that occurs during the sclerotization processes and causes amber coloration. The pigments, similarly to guanine crystals, are not an integral part of the cuticle, but are contained within intracellular granules of the hypodermal cells, called chromophores or melanosomes. Ommochroms, Kynurenine and 3-hydroxykynurenine and melanin are the most common pigments in spiders. They endow the spider with colours ranging from yellow, orange, red, to brown and black [66]. The oxidation state and conjugation with proteins can change the colour appearance. Bilin pigments, consisting of a linear arrangement of pyrroles, give rise to green and blue colours depending on chelated metal ions or other conjugates [66]. Bilins are likely the most common cause for non-structural green colour in spiders [94]. Crab spiders are known for their ability to change their colour according to the background on which they reside [95]. They achieve this by changing ommochrome metabolism leading to change in chromatophore composition, in a slow process called morphological colour change. By changing the guanine distribution within cells, some spiders are able to instantly change their colour when agitated. This instant reaction is termed physiological colour change. In both cases, the result usually leads to increased crypsis [66]. While often pigments are contained intercellularly, they sometimes also occur incorporated into the cuticle itself [66,92,96].

In summary, structural hierarchy gives rise to superimposed optical effects. The organization of lamellae, pores, filaments and gratings at sub-micrometre length scales causes interference with a bandwidth of the reflected light that is determined by the periodicity of the structures. The degree of order or disorder at this length scale will determine the purity (or bandwidth) of the reflected hue. At the micrometre scale, the overall shape of the colour producing structure (setae, scales, etc.) may further affect the degree of iridescence as seen in the peacock spider scales or tarantula setae. Finally, at the macroscopic scale, the spatial organization of setae and scales in organized parallel arrangements or uncorrelated arrays (figure 7) on the spider body affects the overall spectral appearance of the animal (figure 7) [81,82]. All this is based on the cuticular material building blocks described above. The combination of strong scatterers (like guanine) or absorbing pigments in the cuticle of the integument or in its hypodermis extends this basic toolbox and affects the final manifestation of colour.

## 5. Conclusion

The exoskeleton of spiders is made of a few main building blocks only: chitin and proteins, sclerotization agents and water. These are organized hierarchically into a broad range of structural ‘designs’ thus forming a material—the cuticle—with an impressive range of physical properties that support its multifunctional nature. Biological materials such as the spider cuticle and the cuticle of other arthropods can be a source of inspiration for material design. When material properties are to be tuned by the structural arrangement of a small number of components, instead of by composition, they are likely to be more easily recyclable than current materials that are optimized by a large number of chemical components [4]. Multi-scale structuring a material and structural grading may be well used to build lightweight structures, thereby reducing the energy costs for their transport, and potentially also the energy needed for their function without compromising their mechanical properties. On the contrary, in addition to rendering these materials more sustainable, mesoscale structuring of materials, including for example steel and concrete, two of the most important CO_2_ emitter industries, has already been shown to lead to improvement of their mechanical properties such as strength and damage resistance, as seen in biological materials [97–103].

We have not discussed here questions related to the formation of cuticle materials. For spiders, the particular details of cuticle formation have not been studied yet, but a lot more is already known for the assembly of insect cuticle. The formation of parallel and helicoidal fibre arrays in locusts' cuticles involves a self-organization process dictated by the boundary conditions determined by the cells and driven by co-assembly of its main building blocks [104–106]. It will require further study in order to fully understand all the steps of this process, but this effort is likely worthwhile and may lead to new ideas that can be implemented in a sustainable synthesis of new ‘smart’ materials, and exquisitely tuned functional structures made from them.

## Data accessibility

This article has no data.
